# Evidence of the presence of nucleic acids and β-glucan in the matrix of non-typeable *Haemophilus influenzae in vitro* biofilms

**DOI:** 10.1038/srep36424

**Published:** 2016-11-02

**Authors:** Mirian Domenech, Elena Pedrero-Vega, Alicia Prieto, Ernesto García

**Affiliations:** 1 Departamento de Microbiología Molecular y Biología de las Infecciones, Centro de Investigaciones Biológicas (CSIC), Ramiro de Maeztu 9, 28040 Madrid, Spain; 2 CIBER de Enfermedades Respiratorias (CIBERES), Instituto de Salud Carlos III, Monforte de Lemos 3–5, 28029 Madrid, Spain; 3 Departamento de Biología Medioambiental, Centro de Investigaciones Biológicas (CSIC), Ramiro de Maeztu 9, 28040 Madrid, Spain

## Abstract

Non-typeable *Haemophilus influenzae* (NT*Hi*) is a Gram-negative bacterium that frequently colonizes the human nasopharynx; it is a common cause of chronic and recurrent otitis media in children and of exacerbations of chronic obstructive pulmonary disease. To date, no exopolysaccharide clearly contributing to NT*Hi* biofilms has been identified. Consequently, there is some debate as to whether NT*Hi* forms biofilms during colonization and infection. The present work shows that NT*Hi* can form biofilms *in vitro,* producing an extracellular matrix composed of proteins, nucleic acids, and a β-glucan. Extracellular DNA, visualized by immunostaining and using fluorochromes, is an important component of this matrix and appears to be essential in biofilm maintenance. Extracellular RNA appears to be required only in the first steps of biofilm formation. Evidence of a matrix polysaccharide was obtained by staining with Calcofluor white M2R and by disaggregating biofilms with cellulase. Using strain 54997, residues of Glc*p*(1→4) in the NT*Hi* biofilm were confirmed by gas-liquid chromatography-mass spectrometry. Evidence that *N*-acetyl-L-cysteine shows notable killing activity towards *in vitro* NT*Hi* biofilm-forming bacteria is also provided.

Non-typeable (non-encapsulated) *Haemophilus influenzae* (NT*Hi*) is an opportunistic pathogen that colonizes the nasopharynx of some 80% of humans^1^. Colonization promotes the development of disease and produces bacterial reservoirs facilitating person-to-person transmission. NT*Hi* is the main bacterial cause of chronic otitis media (OM) with effusion, recurrent acute OM, and acute OM with treatment failure^2^. In addition, NT*Hi* is one of the main causal agents of upper and lower respiratory tract disease, such as sinusitis, conjunctivitis, and exacerbations of cystic fibrosis (CF) and chronic obstructive pulmonary disease (COPD)^3^. Indeed, chronic infection with NT*Hi* contributes to the progression of COPD and accounts for approximately 20–30% of all exacerbation episodes. It should be noted that, by 2020, COPD is projected to rank fifth in the global burden of disease^4^. In addition, NT*Hi* infections frequently become chronic and recurrent; up to 30% of children who experience at least one episode of OM, re-experience three or more episodes before three years of age^5^.

Chronicity and recurrence are characteristic of diseases produced by biofilm-forming microorganisms^6^; bacterial strains isolated from patients with persistent infections are usually biofilm producers^7^. A biofilm is defined as layers of cells of microorganisms adhered to the surface of an organic or inorganic substrate and embedded in an extracellular matrix^8^. This matrix consists of a mixture of biopolymers (extracellular polymeric substances or EPS) synthesized largely by the biofilm-producing microorganisms themselves. In most cases, the formation of biofilms is controlled by a regulatory switch, and the transition from planktonic to biofilm growth involves the production of an extracellular polysaccharide plus other macromolecules^9^. It has been reported that NT*Hi* strains isolated from patients with CF, OM or COPD are prone to form biofilms *in vitro* and *in vivo*^10^,^11^. In the past, some have expressed doubts about the relevance of NT*Hi* biofilms in disease^12^, although evidence exists that NT*Hi* can grow in an aggregate form that is consistent with a biofilm and that this form of growth affects virulence^9^,^10^. Whether NT*Hi* is truly capable of biofilm formation, however, is a matter of debate^13^. Firstly, while a number of studies have reported quorum sensing in NT*Hi*, issues exist regarding the relationship between this and biofilm formation in these bacteria; for example, NT*Hi* mutants for several quorum sensing genes can still form supposed biofilms^14^. Secondly, while *in vivo* studies suggest extracellular DNA (eDNA) to be a major element of NT*Hi* biofilms^15^, and while treatment with DNase I increases the susceptibility of the NT*Hi* present to certain antibiotics^16^, it is debatable whether this eDNA (or any EPS present) is of bacterial or host origin (or both)^13^. Even if the eDNA were bacterial, it could be the product of autolysis. The purported existence in the matrix of *in vitro* biofilm-specific proteins has, however, been reported providing some evidence that biofilm formation does occur^17^.

In addition to proteins and eDNA, two components of NT*Hi* lipooligosaccharides (LOS) have been reported important in biofilm formation: sialic acid (Neu5Ac) and phosphorylcholine^14^. Since NT*Hi* is auxotroph for Neu5Ac, this compound must be taken up from the host, and mutants deficient in Neu5Ac incorporation into LOS are reported impaired in their capacity to form biofilms *in vitro*^18^. Moreover, a direct relationship between the phosphorylcholine content of the LOS and the capacity to form biofilms has been reported^19^,^20^, although this finding may have been the result of the particular method used to test biofilm formation, i.e., continuous flow or static systems^21^. In most microorganisms, the transition from planktonic to biofilm growth involves production of an extracellular polysaccharide^22^. However, there has yet to be an exopolysaccharide identified that clearly contributes to NT*Hi* biofilms^23^. Thus, the question of whether NT*Hi* really forms biofilms has remained partly unanswered^13^. The present work goes some way to settling this issue by providing evidence of substantial amounts of bacterial eDNA, plus a hitherto unknown extracellular β-glucan polysaccharide, among the EPS components of *in vitro* NT*HI* biofilms.

## Results

### Biofilm formation capacity of different NTHi strains

The biofilm-forming capacity of four NT*Hi* strains, i.e., 54997, 86–028NP, 375 Δ*opsX* and Rd KW20, was examined. It has been reported that strain NT*Hi* 375 Δ*opsX* (a strain deficient in the heptosyltransferase I for lipopolysaccharide biosynthesis) forms biofilms not significantly different to those produced by the wild-type strain^20^. In addition, the genomes of strains 375 and 86–028NP share notable synteny (although they also show distinct genome rearrangements) (Supplementary Fig. S1). This agrees with the finding that the sequence types (ST) of these strains (see Methods) share 5 of the 7 alleles used in multilocus sequence typing.

**Figure 1:**
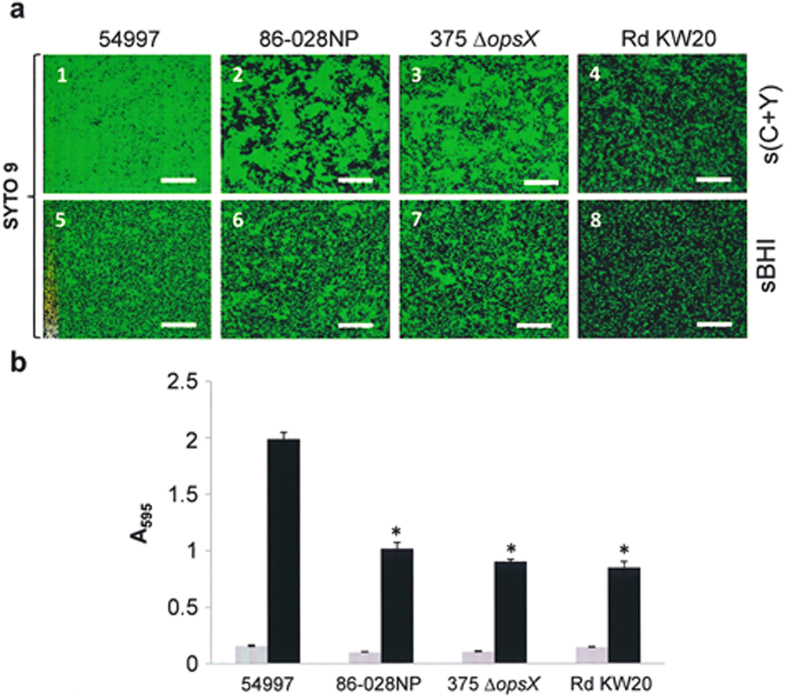
Biofilm formation capacity of four NT*Hi* strains. Bacteria were incubated for 6 h at 37 °C in a 5% CO_2_ atmosphere to allow biofilm development. (**a**) CLSM images of the NT*Hi* strains grown in s(C+Y) and sBHI media. The cells in the biofilms were stained with SYTO 9. Horizontal reconstructions of 55 scans (*x*–*y* plane) are shown. In all images the scale bar = 25 μm. (**b**) For biofilm formation, NT*Hi* cells were grown in s(C+Y) medium on polystyrene microtiter plates and stained with CV. Grey and black bars indicate growth (adherent plus non-adherent cells) and biofilm formation respectively. *P < 0.001 compared to the biofilm formed by strain 54997. The results are the average of three independent experiments each performed in triplicate.

It was observed here that all strains formed supposed biofilms in both C medium supplemented with yeast extract, haemin and NAD [s(C+Y)] (especially well) and in supplemented brain-heart infusion (sBHI) (Fig. 1). The s(C+Y) medium was developed in our laboratory during preliminary experiments aimed at producing *Streptococcus pneumoniae*–NT*Hi* mixed biofilms (unpublished results). Moreover, this medium has the additional advantage that it does not produce a detectable background after crystal violet (CV) staining, unlike sBHI. In both media, however, strains 54997 and Rd KW20 were the best and worst producers respectively. For this reason, strain 54997 was used for most of the following experiments.

### Extracellular proteins and nucleic acids

**Figure 2:**
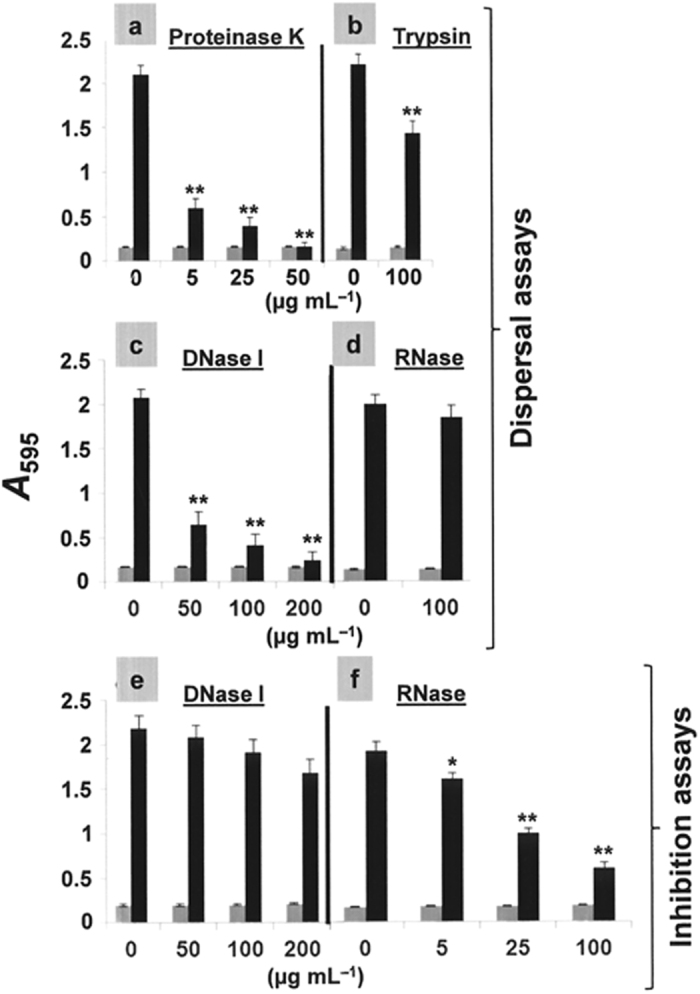
Inhibition and dispersal of NT*Hi* biofilms with proteases and nucleases. Panels **a**–**d** correspond to dispersal assays. After biofilm development (6 h at 37 °C under 5% CO_2_), non-adherent cells were removed, enzymes at the indicated concentrations were added and incubation allowed for an additional 1 h at 37 °C under 5% CO_2_ before staining with CV to quantify biofilm formation. Panels **e** and **f** correspond to inhibition assays. NT*Hi* 54997 was grown overnight at 37 °C under 5% CO_2_ to an *A*_550_ value of 0.5 (corresponding to the late exponential phase of growth) in s(C+Y) medium, centrifuged, and adjusted to an *A*_550_ of 0.6 with fresh medium. The cell suspension was then diluted 100-fold, and 200 μl aliquots were distributed in the wells of a microtiter plate, which was then incubated for 6 h at 37 °C under 5% CO_2_ with DNase I, or RNase at the indicated concentrations. In all panels, grey and black bars indicate growth (adherent plus non-adherent cells) and biofilm formation respectively. *P < 0.05 and **P < 0.001 compared with the untreated control.

Exposure to proteolytic enzymes led to the dispersal of the NT*Hi* biofilms indicating their matrix to contain proteins important in their maintenance (Fig. 2a,b). Treatment of the biofilms with DNase I confirmed eDNA to be present in the matrix, and to be important in its preservation (Fig. 2c). Nuclease treatment of growing biofilms strongly suggested the importance of extracellular RNA (but not DNA) in biofilm formation (Fig. 2e,f). However, once a biofilm formed, it appeared that extracellular RNA was not necessary to ensure its continued integrity (Fig. 2d).

**Figure 3:**
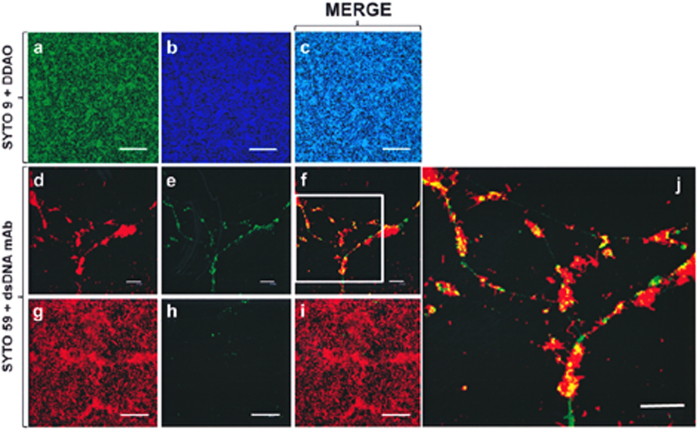
CLSM evidence of eDNA in NT*Hi* biofilms. A biofilm of NT*Hi* 54997 was stained with a combination of SYTO 9 (**a**, green) and DDAO (**b**, blue), or with a combination of SYTO 59 (**d** and **g** red) and anti-dsDNA mouse monoclonal antibody, followed by Alexa Fluor 488-labelled goat anti-mouse IgG (dsDNA mAb) (**e** and **h**) green). Images **d**–**f** and **g**–**i** correspond, respectively, to the top and bottom parts of the biofilm. Images (**c**,**f** and **i**) are mergers of the two previous channels and represent maximum projections of a series of *x*–*y* sections. Scale bars = 25 μm. (**j**) Enlargement of the area marked with a rectangle in (**f**). Yellow indicates co-localisation of the two fluorophores.

*In situ* staining with DDAO (7-hydroxy-9H-[1,3-dichloro-9,9-dimethylacridin-2-one]) revealed abundant, apparently cell-associated eDNA in a 6 h-old biofilm formed by NT*Hi* strain 54997 in s(C+Y) medium (Fig. 3a–c). Moreover, immunostaining with anti-double-stranded (ds) DNA monoclonal antibodies revealed a network-like structure consisting of long DNA strands with numerous bacteria at the top of the biofilm (Fig. 3d–f,j). At the bottom, only small areas of what appeared to be compacted eDNA were seen (Fig. 3g–i). Planktonic cultures of strain 54997 incubated with DDAO, or immunostained with anti-dsDNA antibodies, showed no DNA-related fluorescence (data not shown).

### Polysaccharide component of the NTHi biofilm matrix

To determine whether matrix polysaccharide(s) is required for biofilm preservation, sodium metaperiodate — a mild oxidant for converting the hydroxyl groups [*cis*-glycol] in carbohydrates to reactive aldehyde groups — was added to a biofilm formed by strain 54997. Notable destruction of NT*Hi* biofilms was observed when treated with 40 μg mL^–1^ of this compound (Supplementary Fig. S2).

**Figure 4:**
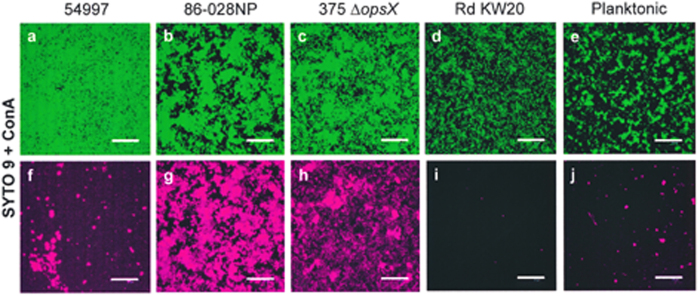
Staining of NT*Hi* biofilms with ConA-Alexa fluor 647. NT*Hi* biofilm matrix stained with SYTO 9 (green fluorescence) (**a**–**d**) and ConA lectin (pink fluorescence) (**f**–**i**). (**e**,**j)** Planktonically-grown 54997 cells stained with SYTO 9 and ConA. Scale bars = 25 μm.

**Figure 5:**
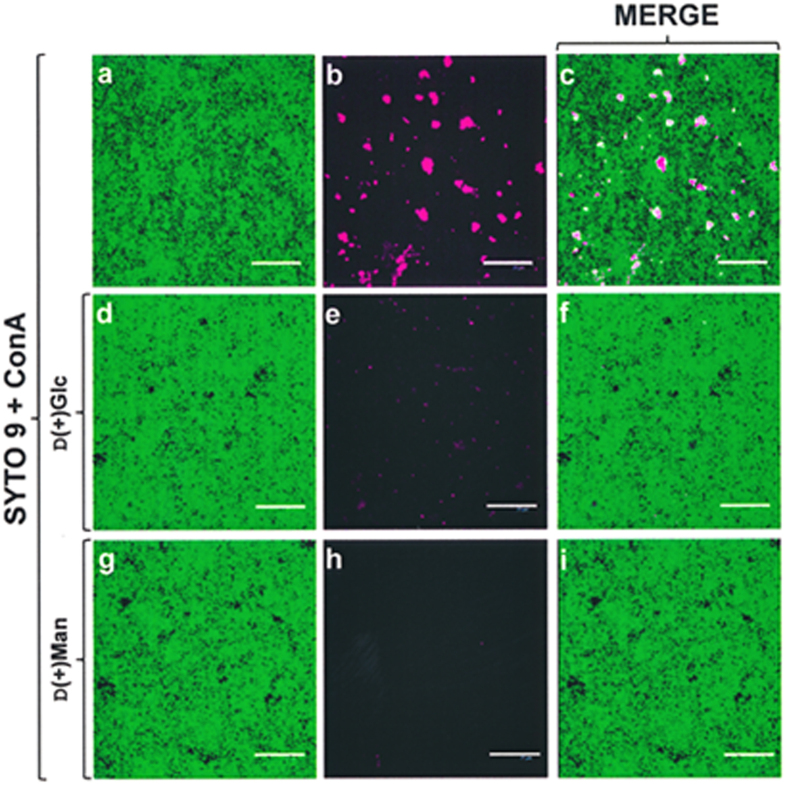
Inhibition of ConA-Alexa fluor 647 staining by monosaccharides. Biofilm of strain 54997 stained with SYTO 9 (**a**; green fluorescence) and ConA lectin (**b**; pink fluorescence). A merger of the above two channels is shown in (**c**). (**d**–**i**) As in (**a**–**c**) but where ConA was incubated with 80 mg mL^‒1^ of D(+)Glc (**d**–**f**) or D(+)Man (**g**–**i**) before staining the biofilm. Scale bars = 25 μm.

In a first attempt to identify the sugar components of the NT*Hi* matrix glycan, five different Alexa-conjugated lectins were used (see Methods). Positive labelling was observed only with concanavalin A (ConA) (Fig. 4). This stained the biofilms formed by NT*Hi* 54997, 86–028NP and 375 Δ*opsX*, but not that formed by strain Rd KW20 (Fig. 4f–i). Labelling intensity varied greatly among strains, suggesting that the biosynthesis of the putative matrix polysaccharide may be strain-dependent. Interestingly, incubation of ConA with 80 mg mL^–1^ of either D(+)glucose (Glc) or D(+)mannose (Man) before addition to the biofilms prevented their labelling, especially when D(+) Man was used (Fig. 5). Planktonically grown cells of all these strains (again with the exception of Rd KW20) also stained with ConA (Fig. 4j and data not shown).

The observation that ConA did not label the biofilm formed by NT*Hi* Rd KW20 suggested the possibility that ConA-labelling might be related to the presence/absence of a pair of high molecular weight (HMW) adhesins described in a number of NT*Hi* isolates^24^. These proteins are synthesized by ≈60% of NT*Hi* isolates, and 90% of these possess two HMW adhesin-coding genes^25^. Interestingly, *hmw* genes are present in strains 86–028NP^26^ and 375^27^, but absent in strain Rd KW20. The adhesin HMW1 (and presumably also HMW2) of strain 12 (also named R2846; Acc. No. CP002276) is a glycoprotein in which 31 asparagine residues are *N*-glycosylated^28^. Based on chemical analysis it has been shown that the carbohydrate of HMW1 contains galactose (Gal), Glc and Man^29^. In the present work, when the amino acid sequence of the HMW1 adhesins of the sequenced strains were aligned and compared to that of strain 12, in which the *N*-glycosylated asparagine residues were originally identified, several changes were found (Supplementary Fig. S3). Five and nine asparagine residues present in strain 12 were not present in strains 86–028NP and 375 respectively. Of these, two and five changes, respectively, were non-conservative substitutions.

**Figure 6:**
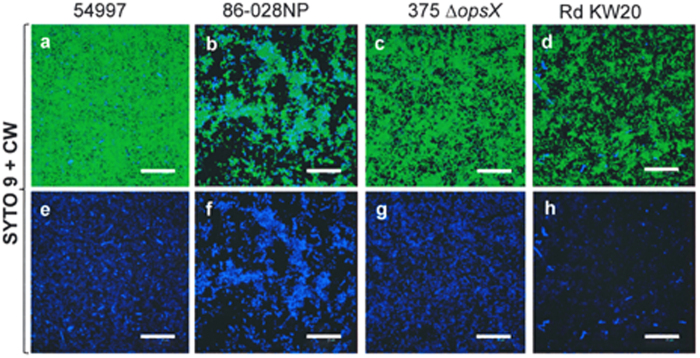
Calcofluor (CW) staining of NT*Hi* biofilms. (**a**–**d**) a merger of biofilms of the four NT*Hi* strains stained with SYTO 9 (green fluorescence) and CW (blue fluorescence). In panels (**e**–**h**) only the CW staining is shown. Scale bars = 25 μm.

**Figure 7:**
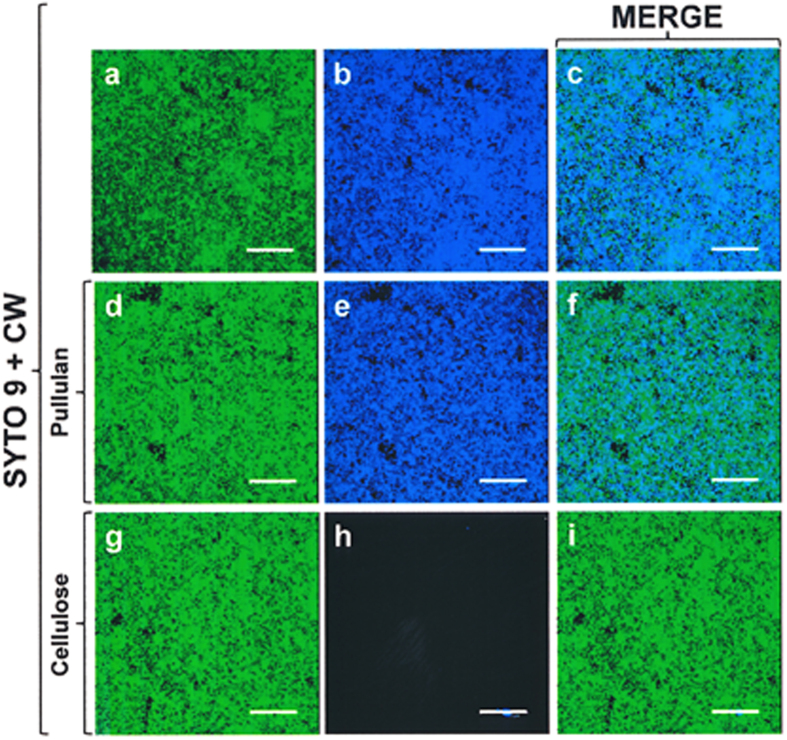
Specificity of Calcofluor (CW) staining. A biofilm of the NT*Hi* 54997 strain stained with SYTO 9 (**a**; green fluorescence) and CW (**b**; blue fluorescence). A merger of the above two channels is also shown (**c**). In panels (**d**–**i**) CW was incubated with pullulan (**d**–**f**) or cellulose (**g**–**i**) (at 25 mg mL^–1^ each), before staining. Scale bars = 25 μm.

Calcofluor white M2R (CW) was used to check for the presence of a glycan component in the NT*Hi* matrix. Biofilm-growing NT*Hi* cells (Fig. 6), but not planktonic cells (not shown), were able to bind CW in significant amounts, and most of the CW-stained material appeared to be cell-associated. Interestingly, the biofilm formed by NTHi 375 Δ*opsX*, a strain lacking all core sugars of the LOS, also stained with CW (Fig. 6g), which strongly suggests that CW labelling of NT*Hi* biofilms is largely unrelated to the presence of LOS components. Since CW is known to bind to β-polysaccharides such as chitin and cellulose^30^, these results suggest the NT*Hi* biofilm to be composed of aggregates of microbial cells encased in an extracellular polysaccharide matrix that contains (at least) β-linked D-glycopyranosyl units. The specificity of CW labelling was checked by pre-incubating the compound with either pullulan (an α-glucan) (Fig. 7d–f) or cellulose (a β-glucan) (Fig. 7g–i). Only cellulose was able to inhibit the binding of CW, as indicated by the loss of fluorescence.

To further investigate the presence of a polysaccharide component in the matrix, NT*Hi* 54997 biofilms were incubated with cellulase. This glycolytic enzyme was very effective in killing the bacteria and dispersing the NT*Hi* biofilms, as determined by *Bac*Light LIVE/DEAD staining (Supplementary Fig. S4). A direct relationship between bacterial killing and biofilm disaggregation could not be established since, at least in some cases, biofilm-growing bacteria may be enzymatically killed without any appreciable biofilm dispersal^31^.

### Chemical analysis of the extracellular polysaccharide

Since, under the present experimental conditions, NT*Hi* 54997 was the best biofilm former (Fig. 1), the extracellular polysaccharide synthesized by biofilm-growing cells of this strain was analyzed by chemical methods. Both the alkali-soluble (AS) and -insoluble (AI) fractions of the biofilm (AS-B and AI-B respectively) were subjected to acid hydrolysis to release the monosaccharides. The same procedure was performed to investigate the planktonic component of the biofilm (i.e., the non-adherent cells in biofilm culture [BP], and planktonic culture [P]). Irrespective of their origin, only small amounts of carbohydrates (<2% of the dry weight) were found in the AS fractions (data not shown) so were studied no further. In contrast, the major monosaccharide component of the AI-B and AI-BP fractions was Glc (up to 40% and 20%, respectively); only minor amounts (4.5% and <1%, respectively) of Man and glucosamine (<0.5% in the AI-B fraction) were detected. No putative LOS components were detected. The AI-P fraction contained 4–5% Man and only <1% Glc (data not shown). The Man component found in the AI fraction may originate from the yeast extract component of the growth medium — s(C+Y) — which contains mannans in large amounts.

**Figure 8:**
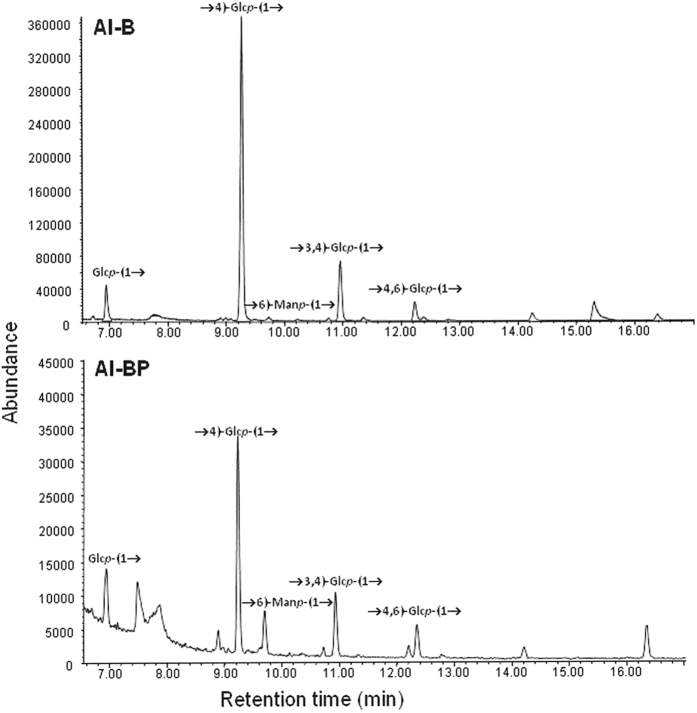
GC-MS chromatograms showing the linkage types identified in the polysaccharides recovered from alkali-insoluble extracts of biofilms (AI-B) and their planktonic component (AI-BP).

Methylation analysis of AI-B and AI-BP showed both fractions to contain the same type of polymer — although it was much more abundant in adherent, biofilm-forming cells (see above) — and to be mainly composed of linear →4)-Glc*p*-(1→ units (Fig. 8). This glucan appears to be slightly branched, as deduced from the presence of peaks identified as terminal glucopyranose residues (Glc*p*-(1→) and branching points at positions *O-*3 and/or *O*-6 on the main chain (Fig. 8). Alkali insolubility^32^, labelling with CW and biofilm destruction with cellulase indicated these units to be connected by β-linkages. Thus, the polysaccharide must be a β-(1 → 4)-glucan.

### Prevention of biofilm formation and therapy

**Figure 9:**
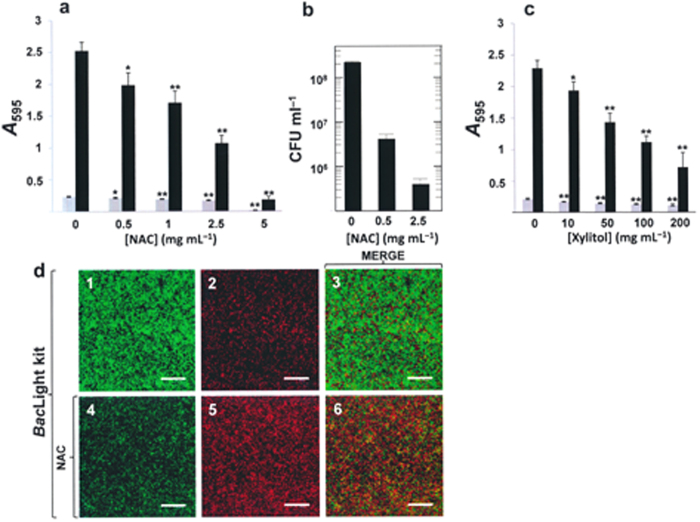
Inhibition of biofilm development in NT*Hi* cultures in the presence of *N*-acetyl-L-cysteine (NAC) or xylitol. (**a**) Strain 54997 was distributed in the wells of a microtiter plate, which was then incubated for 6 h at 37 °C under 5% CO_2_ in the presence of different concentrations of NAC. (**b**) Killing of NT*Hi* 54997 cells growing as biofilms by incubation at 37 °C under 5% CO_2_ for 90 min with NAC at the indicated concentrations. (**c**) As in (**a**), but with xylitol instead of NAC. Biofilm formation was quantified by staining with CV. In panels **a** and **c**, grey and blackened bars indicate growth (adherent plus non-adherent cells) and biofilm formation respectively. Results represent the mean ± standard error of at least four independent experiments, each performed in triplicate. *P < 0.01 and **P < 0.001 compared with the control. (**d**) CLSM image of the viability of biofilm-grown NT*Hi* 54997 untreated (panels 1–3) or treated (panels 4–6) with 0.5 mg ml^–1^ of NAC for 90 min at 37 °C under 5% CO_2_. Cells in the biofilms were stained with the *Bac*Light kit showing viable (green fluorescence) and non-viable (red fluorescence) bacteria. Images are horizontal three-dimensional reconstructions of 25 scans in the *x*–*y* plane. Scale bars = 25 μm.

Drug ‘repurposing’ (or ‘reprofiling’) appears a promising possibility for speeding up drug discovery, reducing failure rates and the associated costs^33^. In the present work, the mucolytic compound *N*-acetyl-L-cysteine (NAC) and the well-known sugar substitute xylitol were examined as candidates for use in future strategies aimed at preventing and improving the management of upper and lower respiratory tract diseases caused by biofilm-forming NT*Hi*. Figure 9a shows that, in the present work, NAC inhibited biofilm formation by NT*Hi* 54997, and caused the death of ≥95% of bacteria in the biofilm when used at concentrations of ≥0.5 mg mL^–1^ (Fig. 9b,d) (well below the minimum inhibitory concentration [MIC] of 2.5 mg mL^–1^; see Methods). Besides, a significant reduction in growth and in biofilm formation by NT*Hi* 54997 was observed when xylitol was used at concentrations of ≥10 mg mL^–1^ (Fig. 9c).

## Discussion

The ability of bacteria to produce biofilms appears to be governed by many genes and to be under tight regulation; certainly, different NT*Hi* isolates show different biofilm-forming capacity^34^ (Fig. 1), perhaps a consequence of the well-known genetic heterogeneity of NT*Hi* populations^35^. Proteins are important components of the biofilm EPS, and different experimental approaches have been employed to try to identify NT*Hi* biofilm-specific proteins. Several proteins are present among the EPS of *in vitro* NT*Hi* biofilms, namely, the adhesins Hap and HMW1/HMW2, and the IgA1 protease^36^. The requirement for the surface protein Hap in biofilm formation has, however, recently been questioned^37^. In an independent study involving liquid chromatography coupled with tandem mass spectrometry, more than 200 proteins were identified in the extracellular matrix of NT*Hi* biofilms formed on Millipore filters^38^. Unfortunately, the presence of lysed-cell components among the samples analyzed could not be ruled out^38^. More recently, mild sonication was used to extract the EPS of *in vitro* NT*Hi* biofilms, and chemical analysis, nuclear magnetic resonance and Fourier transformed infrared spectroscopy used to reveal the presence of proteins (18 of which were proposed potential biofilm-specific proteins), polysaccharide(s) and DNA among them^17^. Whether only intact bacteria were the source of these macromolecules could not, however, be conclusively demonstrated. No protein identification was attempted in the present study, but treatment with either proteinase K or trypsin fully confirmed proteins to be required for the maintenance of NT*Hi* biofilms.

Nucleic acids are also important components of NT*Hi* biofilm matrices. Extracellular RNA appears to be required for the initial attachment of — but not the maintenance of — a biofilm. Certainly, the presence of RNase led to a significant reduction in biofilm development (Fig. 2f), but had no effect on already formed biofilms (Fig. 2d). To our knowledge, the requirement of extracellular RNA for biofilm formation has never before been reported, although this result may not be representative of all NT*Hi* clinical isolates. Although the release of RNA to the medium is normally attributed to autolytic processes, *Mycobacterium tuberculosis* and *Escherichia coli* do secrete small RNA fragments into the culture medium in the absence of detectable autolysis^39^,^40^. In contrast, the importance of eDNA in both the establishment and maintenance of bacterial biofilms is well known.

The eDNA of NT*Hi* and other bacterial biofilms can be visualized under the confocal laser scanning microscope (CLSM) using fluorescent, ds specific stains^15^ plus propidium iodide, ethidium bromide, 4′,6-diamidino-2-phenylindole (DAPI) or SYTO dyes. The sensitivity of the microscope must, however, be strongly increased, and good quality images can be hard to obtain^41^. Recently, DDAO has been shown very suitable for selectively targeting eDNA given its enhanced fluorescent properties and since its molecular size prevents the stain from penetrating intact cell membranes^42^. In the present work, staining with DDAO showed eDNA to be distributed throughout the biofilm but, when anti-dsDNA antibodies were present, long filaments of eDNA with attached bacteria were evident mostly in the upper part of the biofilm (Fig. 3). Since most were actively growing NT*Hi* cells, it would appear unlikely that eDNA fibres are formed exclusively via autolysis. Rather, some kind of programmed release, involving a biofilm-specific secretion process, might be at work, although further research is needed to test this hypothesis. Previous studies have shown that eDNA binds extracellular proteins such as PilA (the type IV pilin protein)^15^ and the bacterial DNABII family of proteins (also known as histone-like proteins or Hlps)^43^. Recent results from our laboratory have shown that the choline-binding proteins of *S. pneumoniae* have the unexpected capacity to strongly bind DNA through electrostatic interactions; they may therefore be important in the early stages of biofilm formation^31^,^44^. This might also be true for other bacteria.

It is generally accepted that carbohydrates are important components of biofilm matrices^45^. The biosynthesis of alginate by species of *Pseudomonas* and *Azotobacter*, and of poly-β-1,6-*N*-acetylglucosamine by many Gram-positive and Gram-negative bacteria, has been quite well studied^22^. However, no exopolysaccharide has been identified that clearly contributes to NTHi biofilms^23^. Sodium metaperiodate has been extensively used to test for the presence of carbohydrates in EPS^46^. This compound induced disaggregation of NT*Hi* biofilms strongly suggesting that they contain a glycan. Moreover, with the notable exception of strain Rd KW20, the incubation of biofilms with ConA revealed the presence of accessible α-Man and/or Glc residues. Although direct evidence is lacking, the absence of the two genes coding for the HMW adhesins (glycoproteins) in strain Rd KW20 suggests that ConA binds to the *N*-linked glycan of HMW. The differences seen in ConA-labelling between the different biofilms may be due to variation in the number of glycosylated Asn residues and/or to variations in the expression of *hmw* genes, which is known to be modulated by phase variation^47^. In any event, as planktonically grown cells of all the NT*Hi* strains tested here (with the exception of Rd KW20) also stained with ConA, the ConA-labelled carbohydrate(s) appears to be not biofilm-specific.

The literature contains no report of any exopolysaccharide in the matrix of NT*Hi* biofilms^23^; the CW staining results provide the first experimental evidence that a cellulose-like β-polysaccharide is present (Fig. 6). Moreover, disaggregation by cellulase, and the chemical analysis of the alkali-insoluble fraction of strain 53997 biofilms, provided compelling evidence of the presence of a cellulose-like carbohydrate with β-(1→4) linked glucosyl residues (and possibly other monosaccharides as putative side chain substituents) among the EPS (Fig. 8). This glucan would appear completely unrelated to glycogen (an α-glucan), which is overproduced when NT*Hi* biofilms are treated with sub-inhibitory concentrations of β-lactam antibiotics^48^.

Cellulose is the most abundant biopolymer on Earth, and is synthesized by bacteria, protists, algae, plants and even by some tunicates. Members of the classes α-, β- and γ-*Proteobacteria* synthesize cellulose as an EPS component, and it plays important roles in biofilm formation and maintenance^49^. However, cellulose production genes have not been found among the members of *Pasteurellaceae*; the present finding of a polysaccharide similar to cellulose in NT*Hi* biofilms was therefore totally unexpected. It has been shown, however, that *Histophilus somni*, a relative of *H. influenzae*, synthesizes an exopolysaccharide composed of a D-mannan polymer and with occasional Gal residues present on side chains during biofilm formation^50^, although the glycosyltrasferase(s) responsible for its synthesis has not been identified. *Aggregatibacter actinomycetemcomitans*, another member of *Pasteurellaceae,* also forms biofilms containing an extracellular homopolymer of *N*-acetylglucosamine residues in β(1 → 6) linkage that acts as an important virulence factor^51^.

A search of the CAZy database^52^ revealed the existence of up to 30 putative glycosyltransferase-coding genes in different NT*Hi* genomes, most of them putatively involved in glycogen production or LOS biosynthesis^53^. Interestingly, the putative glycosyltransferases LsgC, LsgE and LsgF of NT*Hi* appear to be homologous (*E* value ≤ 10^–15^), respectively, to AmsD, AmsB and AmsE, proteins involved in the synthesis of amylovoran, the acidic exopolysaccharide of *Erwinia amylovora*^54^. These corresponding NT*Hi* genes are part of the *lsg* locus (from HI_1695 to HI_1700 in the Rd KW20 chromosome) and are fully conserved across NT*Hi* isolates. Further studies are warranted to determine whether the *lsg* gene products play a role in the synthesis and/or transport of the biofilm-specific β-glucan described in this study.

One of the most important and persistent problems posed by biofilms is the tolerance bestowed upon the communities they house to antibiotic therapy and host defence mechanisms. To our knowledge, the literature contains only a few reports on the activity of antibiotics against NT*Hi* biofilms; these have involved the use of single and multiple antibiotics such as quinolones, macrolides, aminoglycoside, penicillin and cephems^55^. The exposure of NT*Hi* biofilms to sub-inhibitory concentrations of β-lactam antibiotics has been reported to produce a strain- and antibiotic-dependent increase in biofilm formation^48^. Certainly, the need for alternatives to antibiotic treatment is becoming ever clearer since bacteria in a biofilm can survive antibiotic concentrations up to 1000 fold those that would kill them when in a planktonic state^56^. Enzymes that degrade the biofilm matrix, inhibitors of quorum-sensing signals, anticoagulant agents, surfactants, and specific bacteriophages and their endolysins may all provide alternatives^57^,^58^,^59^. NAC, a thiol-containing antioxidant that disrupts disulphide bonds in mucus, has been clinically available for several decades and is used in the treatment of a variety of clinical conditions including chronic bronchitis, ototoxicity caused by certain anti-cancer (*e.g.,* cisplatin) or antibacterial (*e.g*., aminoglycosides) drugs, and in acetaminophen (paracetamol) poisoning^60^. However, it also has antibacterial properties, particularly against microorganisms (Gram-positive and Gram-negative) growing in biofilms^61^. Interestingly, NAC has been reported not only to inhibit the *in vitro* formation of *S. pneumoniae* biofilms, but also to disaggregate already formed biofilms^62^. The concentrations of NAC that inhibited and killed the bacteria of the NT*Hi* biofilms are similar to those that are theoretically obtained in oropharyngeal secretions during normal oral NAC treatment with 200–600 mg tablets taken two or three times daily^63^. Higher doses (up to 2400 mg/day divided into two doses over 30 days) are also well tolerated and beneficial in patients with CF^64^. Moreover, if required, NAC can be administered directly into the middle ear. In fact, transtympanic injections of up to 20 mg mL^–1^ NAC appear to be well tolerated in humans, as demonstrated in a recent clinical trial^65^.

The polyalcohol xylitol may have a variety of medical and pharmaceutical applications, including the treatment and/or prevention of acute OM^66^,^67^ and viral respiratory diseases^68^. It is reported that xylitol evades microbial resistance and can control infection both alone and in combination with other compounds. Moreover, it has been documented that xylitol has anti-adherent properties that may be relevant for fighting biofilm formation^69^. In the present *in vitro* system, xylitol was indeed capable of inhibiting the formation of NT*Hi* biofilms, but at concentrations much higher than those required by NAC for an equivalent reduction (Fig. 9).

In conclusion, this study shows that, actively growing NT*Hi* cells release eDNA (a major EPS component), and that the biofilm produced contains a hitherto unknown β-glucan. Together with our previous results^31^,^44^, plus those of other authors on pneumococcal biofilms^70^, the present findings pave the way for detailed *in vitro* studies on more complex pneumococcal–NT*Hi* biofilms.

## Methods

### Bacterial strains, growth conditions, biofilm formation and susceptibility testing

Four NT*Hi* strains were used: 1) 54997, isolated from a patient with acute OM^11^; 2) 86–028NP (ST33), recovered from the nasopharynx of a child with chronic OM^26^; 3) a Δ*opsX* mutant of strain 375 (ST3)^27^ that synthesizes a truncated LOS lacking all core sugars and exposing the 3-deoxy-α-D-manno-octulosonic acid attached to lipid A^20^; and 4) Rd KW20 (ST47), a mutant of a serotype d strain, which completely lacks the entire capsule locus due to a deletion^71^. Unless otherwise stated, NT*Hi* strains were grown at 37 °C in a 5% CO_2_ atmosphere in C+Y medium^31^ or brain-heart infusion (BHI) supplemented with 10 μg mL^–1^ each of haemin and NAD, referred to as s(C+Y) and sBHI respectively. Both media were supplemented with NAD and haemin to allow the growth of *H. influenzae*. When required, NT*Hi* was grown on chocolate agar plates (bioMérieux).

For biofilm formation, NT*Hi* strains were grown at 37 °C and under 5% CO_2_ in sBHI medium to an absorbance at 550 nm (*A*_550_) of 0.5. They were then sedimented by centrifugation, resuspended in an equal volume of s(C+Y) or sBHI, and diluted 100-fold. Inocula of 4–5 × 10^6^ colony-forming units (CFU) mL^−1^ were dispensed into each well of Costar 3595 96-well polystyrene microtiter plates (Corning). Plates were incubated at 37 °C for 5–6 h in a 5% CO_2_ atmosphere, and bacterial growth (adherent plus non-adherent bacteria) was determined by measuring the *A*_595_ using a VERSAmax microplate absorbance reader (Molecular Devices). Biofilm formation was measured using a modified CV assay^72^. Fifty microlitres of a 1% solution of CV were added to each well. The plates were then incubated at room temperature for approximately 15 min, rinsed three times with 200 μL of distilled water, and air dried. CV-stained biofilm formation was quantified by solubilizing the biofilm with 95% ethanol (200 μL/well) and then determining the *A*_595_. For the inhibition of biofilm formation, the enzymes or antioxidants to be tested were added to the bacteria at the beginning of the incubation in the plates. For dispersal of biofilms, after biofilm formation for 6 h, non-attached cells were withdrawn, the enzymes or antioxidants were added and incubated for 1–1.5 h at 37 °C.

The susceptibility of NT*Hi* isolates to antibacterial agents was determined using the broth microdilution method according to CLSI guidelines^73^. The MIC values for NAC and xylitol were identical (2.5 mg mL^–1^) (data not shown).

### Microscopic observation of biofilms

For the observation of NT*Hi* biofilms by CLSM, strains were grown on glass-bottomed dishes (WillCo-dish, WillCo Wells) for 5–6 h at 37 °C in a 5% CO_2_ atmosphere. Following incubation, the culture medium was removed and the biofilm rinsed with sterile water to remove non-adherent bacteria. The biofilms were then stained with DDAO (H6482, Invitrogen), anti-dsDNA antibody (ab27156, Abcam) (at 2–25 μg mL^–1^ each), and SYTO 9 (10 μM) (S34854, Invitrogen), SYTO 59 (10 μM) (S11341, Invitrogen) and CW (50 μg mL^–1^) (#18909, Sigma-Aldrich). When indicated, biofilms were stained with the bacterial viability *Bac*Light kit (5 μM) (L7007, Invitrogen). To tentatively identify the sugar components of the NT*Hi* matrix glycan, Alexa-conjugated lectins, i.e., ConA (specific for α-Man and α/β-Glc), peanut agglutinin (PNA; specific for terminal residues of β-Gal, e.g., in Galβ-1,3-*N*-acetylgalactosamine [GalNAc] residues of *N*-glycans and glycolipids), soybean agglutinin (SBA; specific for GalNAc as in GalNAcα-1,3-Gal of *O*-linked glycopeptides), wheat germ agglutinin (WGA; specific for *N*-acetylglucosamine and Neu5Ac) and *Helix pomatia* agglutinin (HPA; specific for α-GalNAc) were used^74^. The biofilms were stained with ConA-Alexa fluor 647 (C21421, Invitrogen), HPA-Alexa fluor 488 (L11271, Invitrogen), PNA-Alexa fluor 594 (L32459, Invitrogen), SBA-Alexa fluor 488 (L11272, Invitrogen) or WGA-Alexa fluor 488 (W11261, Invitrogen) at 5–25 μg mL^–1^ each. All staining procedures involved incubation for 10–20 min at room temperature in the dark, except when biofilms were incubated with mouse anti-dsDNA antibody (2 μg mL^–1^); this involved 1 h incubation at 4 °C followed by 30 min incubation at room temperature in the dark with Alexa fluor 488-labelled goat anti-mouse IgG (1:500) (A-11001, Invitrogen) (diluted 1/500). Lectins and CW were incubated for 20 min with 25–80 mg mL^−1^ of specific sugars or polysaccharides at room temperature in the dark to check their binding^75^. After staining, the biofilms were gently rinsed with 0.5 ml PBS. Observations were made at a 63× magnification using a Leica TCS-SP2-AOBS-UV CLSM equipped with an argon ion laser. Images were analyzed using LCS software from Leica. Projections were obtained in the planes *x*–*y* (individual scans at 0.5 μm intervals) and *x*–*z* (images at 6 μm intervals).

### Analysis of extracellular and cell surface-associated glycans

EPS were prepared by growing NT*Hi* 54997 in 50 Petri dishes (10 cm diameter), each containing 20 mL of s(C+Y) medium, at 37 °C for 6 h under 5% CO_2_ without shaking, or in 1 L of the same medium under planktonic (P) conditions, i.e., in a culture flask. The non-adherent cells in the dishes (BP) were pipetted off and the biofilm-grown cells (B) suspended in 20 mM sodium phosphate buffer, pH 7.0. The cells were then treated with NaOH (1 M, final concentration) as described elsewhere^31^. In short, alkali-soluble (AS) and alkali-insoluble (AI) fractions were dialyzed using membranes with a molecular mass cut-off of 3.5 kDa (SnakeSkin™ Pleted Dialysis Tubing; Thermo Scientific) and the different products were freeze-dried. To determine their monosaccharide composition, the AS and AI fractions were acid-hydrolyzed. The products were reduced with sodium borohydride and the corresponding alditols acetylated. Identification and quantification were performed by gas-liquid chromatography-mass spectrometry (GC-MS). For quantification, *myo*-inositol (100 μg) was used as an internal standard. To analyze the bonding between the monosaccharide components, 1–3 mg of the AI and AS fractions were permethylated, hydrolyzed and converted into a mixture of partially methylated alditol acetates that was analyzed by GC-MS. Quantifications were made according to the peak area.

### Statistical analysis

Data comparisons were performed using the two-tailed Student *t*-test.

## Additional Information

**How to cite this article**: Domenech, M. *et al*. Evidence of the presence of nucleic acids and β-glucan in the matrix of non-typeable *Haemophilus influenzae in vitro* biofilms. *Sci. Rep.* **6**, 36424; doi: 10.1038/srep36424 (2016).

**Publisher’s note**: Springer Nature remains neutral with regard to jurisdictional claims in published maps and institutional affiliations.

## Supplementary Material

Supplementary Information
