# Supplementary material for: Evidence of the presence of nucleic acids and β-glucan in the matrix of non-typeable Haemophilus influenzae in vitro biofilms
Source: Sci Rep. 2016 Nov 2;6:36424. doi: 10.1038/srep36424 (PMC5090351; doi:10.1038/srep36424)
Supplement: Supplementary Information [file srep36424-s1.pdf]

Supplementary Information

Evidence of the presence of nucleic acids and β-glucan in the matrix of non-typeable *Haemophilus influenzae in vitro* biofilms

Mirian Domenech[1,3], Elena Pedrero-Vega[1], Alicia Prieto[2] and Ernesto García[1,3]

[1]Departamento de Microbiología Molecular y Biología de las Infecciones, and 2Departamento de Biología Medioambiental, Centro de Investigaciones Biológicas (CSIC), Ramiro de Maeztu 9, 28040 Madrid, Spain. [3]CIBER de Enfermedades Respiratorias (CIBERES), Instituto de Salud Carlos III, Monforte de Lemos 3–5, 28029 Madrid, Spain.

Correspondence to: Mirian Domenech [mirid@cib.csic.es; Tel. (+34) 918 373 112; Fax (+34) 915 360 432].

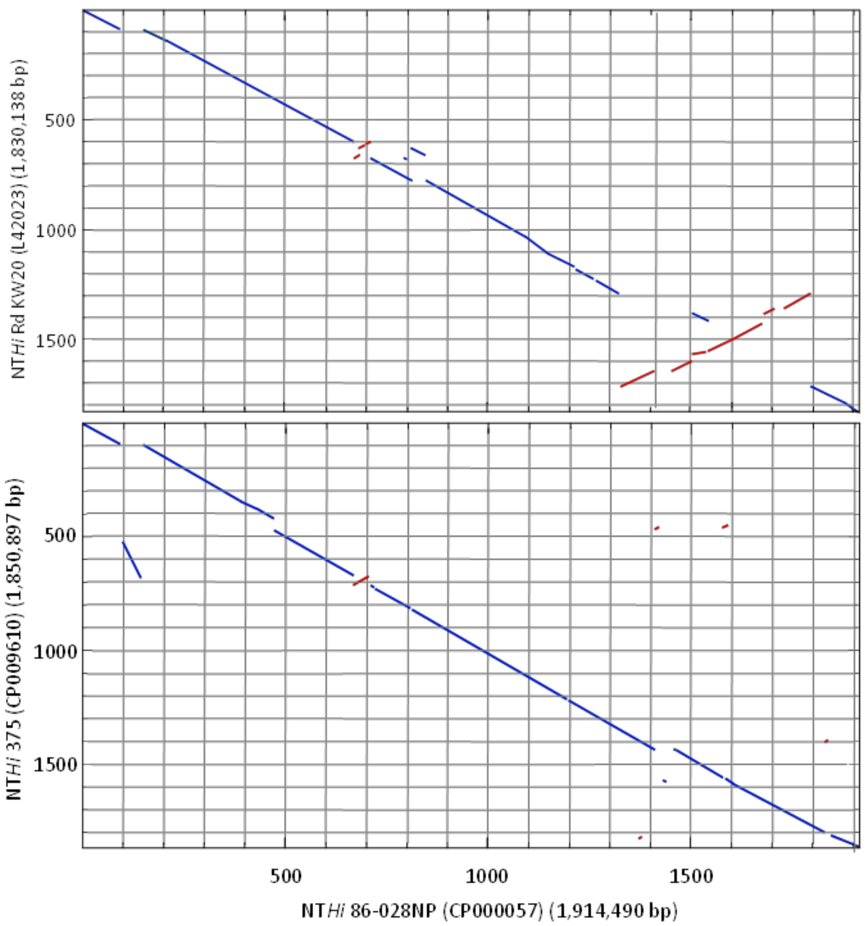

Supplementary Figure S1. **Dot slot matrices calculated for the genome sequences of three non-encapsulated *H. influenzae* strains.** The genome alignment of strains Rd KW20 and 86-028NP served as a control[1]. As nucleotide position 1 of the genomic sequence of strains Rd KW20 and 86-028NP corresponds to nucleotide 1,099,336 of the reverse complement of that of strain 375 (Hi375) (Acc. No. CP009610)[2], the latter sequence was rearranged accordingly to match the genome of the other two strains. The dot matrices were calculated using wgVISTA software[3]. The diagonal lines in the image show the homologous regions between the compared genomes. Blue and red lines indicate that the regions are on the same strand, or on opposite strands, respectively.

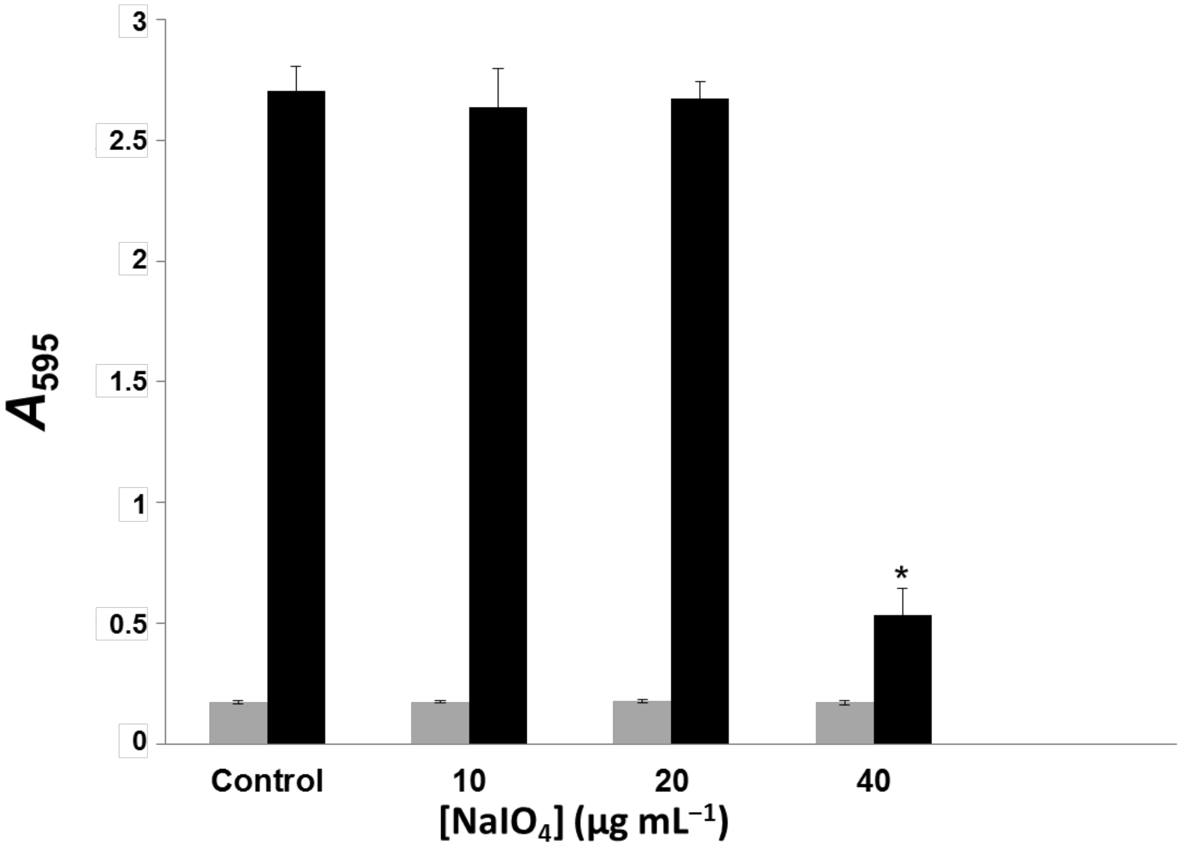

Supplementary Figure S2. **Disaggregation of a NT*Hi* 54997 biofilm by $NaIO_4$.** Cells of NT*Hi* 54997 were distributed in the wells of a microtiter plate, which was then incubated for 7 h at 37 °C in a 5% CO_2 atmosphere. Grey bars indicate growth (adherent plus non-adherent bacteria). Biofilms were washed with sterile water and incubated with $NaIO_4$ for 2 h at 37 °C under 5% CO_2. Biofilm formation was quantified by staining with CV (black bars). *P < 0.001 compared to the control.

a

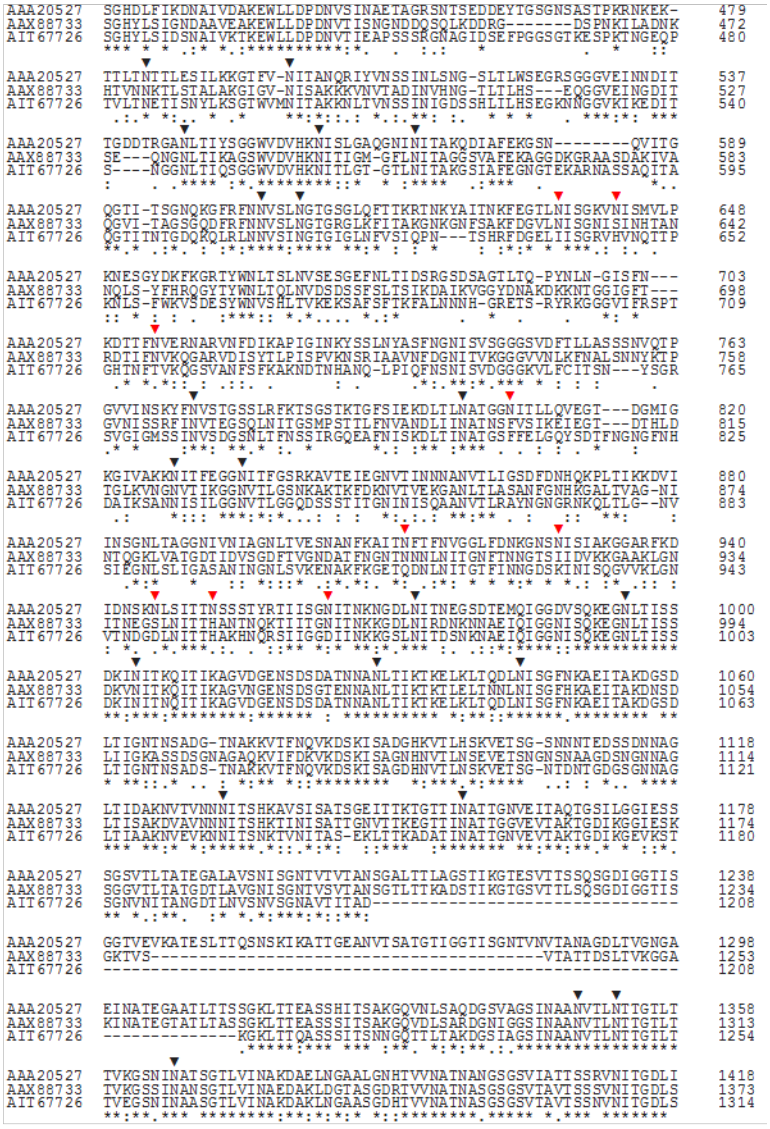

b

Strain 12 (AAA20527)	Strain 86-028NP (AAX88733)	Strain Hi375 (AIT67726)
N636	N630	I640
N642	S636	H646
N709	N704	T715
N806	F801	F808
N912	N906	Q915
N928	I922	K931
N946	S940	D949
N952	H946	H955
N964	N958	D967

Supplementary Figure S3. **Localization of *N*-glycosylated Asn residues in three different HMW1 adhesins of NT*Hi*.** (**a**) Multiple partial alignment of the HMW1 adhesins of strains 12 (AAA20527), 86-028NP (AAX88733) and 375 (AIT67726). The locations of *N*-glycosylated Asn residues were taken from a previous publication[4]. Black and red triangles point to *N*-glycosylated Asn residues conserved and not conserved, respectively, in the three aligned proteins. (**b**) shows the Asn residues of HMW1 of strain 12 in comparison with those of the other two strains. Conservative and non-conservative amino acid substitutions are shown in a grey and black background respectively.

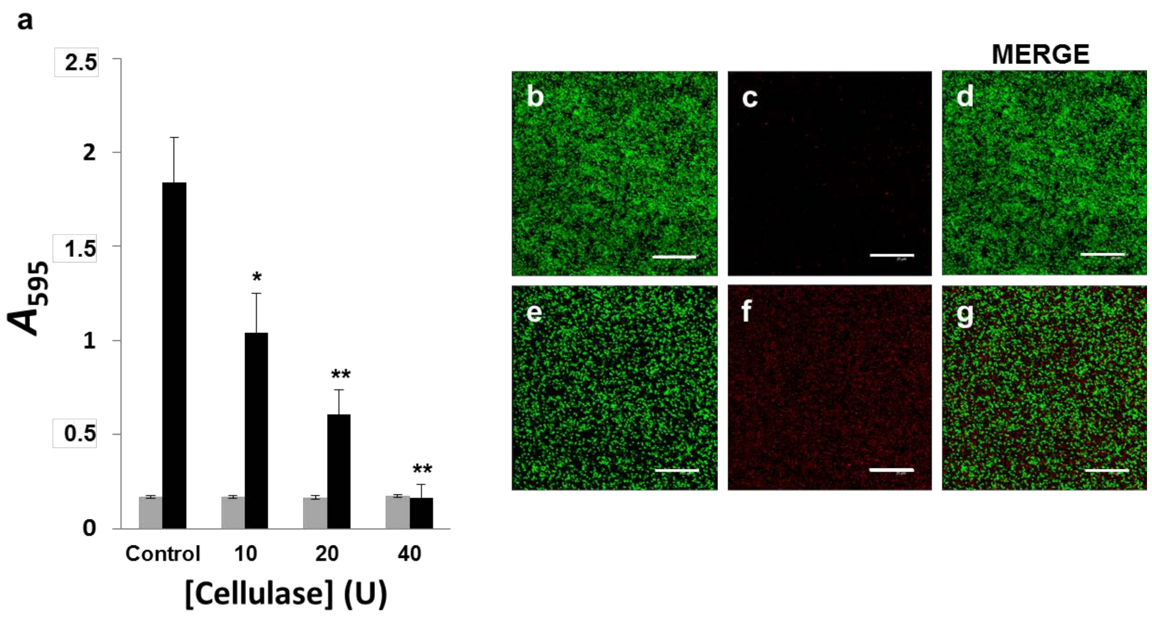

Supplementary Figure S4. **NT*Hi* 54997 biofilms treated with cellulase.** Cells of NT*Hi* 54997 were distributed in the wells of a microtiter plate which was incubated for 6 h at 37 °C in a 5% CO_2 atmosphere. Grey bars indicate growth (adherent plus non-adherent bacteria). (**a**) The medium was withdrawn and cellulase (dissolved in 50 mM sodium citrate buffer, pH 4.6) added. The final volume was adjusted with distilled water to 200 μl and incubation was continued for 90 min at 37°C under 5% CO_2. A control biofilm (without enzyme) was incubated under identical conditions. Biofilm formation was quantified by staining with CV (black bars). **P* < 0.01 and ***P* < 0.001 compared to the control. (**b**–**g**) CLSM images of NTHi biofilms. NTHi 54997 biofilms were untreated (**b**–**d**) or treated (**e**–**g**) with 40 U mL^{-1} of cellulase for 90 min at 37 °C in a 5% CO_2 atmosphere. After treatment, the cells in the biofilms were stained with the *Bac*Light LIVE/DEAD kit to reveal viable (green fluorescence) and non-viable (red fluorescence) bacteria. In all images the scale bar = 25 μm.

Supplementary references

1. Harrison, A. *et al.* Genomic sequence of an otitis media isolate of nontypeable *Haemophilus influenzae*: comparative study with *H. influenzae* serotype d, strain KW20. *J. Bacteriol.* **187**, 4627–4636 (2005).
2. Mell, J. C. *et al.* Complete genome sequence of *Haemophilus influenzae* strain 375 from the middle ear of a pediatric patient with otitis media. *Genome Announc.* **2**, e01245-01214 (2014).
3. Frazer, K. A., Pachter, L., Poliakov, A., Rubin, E. M. & Dubchak, I. VISTA: computational tools for comparative genomics. *Nucleic Acids Res.* **32**, W273–W279 (2004).
4. Gross, J. *et al.* The *Haemophilus influenzae* HMW1 adhesin is a glycoprotein with an unusual *N*-linked carbohydrate modification. *J. Biol. Chem.* **283**, 26010–26015 (2008).
